# Effect of Supplementary Materials on the Autogenous Shrinkage of Cement Paste

**DOI:** 10.3390/ma13153367

**Published:** 2020-07-29

**Authors:** Tianshi Lu, Zhenming Li, Hao Huang

**Affiliations:** Department of Materials, Mechanics, Management & Design, Faculty of Civil Engineering and Geoscience, Delft University of Technology, Mekelweg 5, Delft 2628, The Netherlands; lutianshi2017@gmail.com (T.L.); H.Huang-1@tudelft.nl (H.H.)

**Keywords:** autogenous shrinkage, silica fume, fly ash, blast furnace slag, cement paste

## Abstract

In recent years more and more attention has been given to autogenous shrinkage due to the increasing use of high-performance concrete, which always contains supplementary materials. With the addition of supplementary materials—e.g., fly ash and blast furnace slag—internal relative humidity, chemical shrinkage and mechanical properties of cement paste will be affected. These properties significantly influence the autogenous shrinkage of cement paste. In this study, three supplementary materials—i.e., silica fume, fly ash and blast furnace slag—are investigated. Measurements of final setting time, internal relative humidity, chemical shrinkage, compressive strength and autogenous deformation of the cement pastes with and without supplementary materials are presented. Two water-binder ratios, 0.3 and 0.4, are considered. The effects of different supplementary materials on autogenous shrinkage of cement paste are discussed.

## 1. Introduction

In recent years, high-performance concrete, which always contains supplementary cementitious materials, has been increasingly used in practice. High-performance concrete not only presents high mechanical strength but also exhibits large autogenous shrinkage [1]. Autogenous shrinkage is one of the major causes of cracking in concrete structures. Early-age cracking of concrete may occur when autogenous shrinkage is restrained. Cracks accelerate the ingress of water and corrosive agents and can impair the overall performance of concrete.

Many studies of autogenous shrinkage have been done during the past few decades [2,3]. There is a general agreement about the existence of a relationship between relative humidity change and autogenous deformation of cement paste [4,5,6,7]. As hydration progresses, due to the consumption of moisture from the pore system, self-desiccation occurs in cement paste and the internal relative humidity drops. The internal driving forces of autogenous shrinkage, which are related to relative humidity development. Disjoining pressure, change in the surface tension of the solid gel particles and capillary tension are considered as the three principal internal driving forces of autogenous shrinkage [8,9,10,11]. Besides internal relative humidity, stiffness of cement paste is another important influencing factor on autogenous shrinkage. Stiffness of cement paste reflects the resistance of cement paste to deformation. With the same internal driving force, cement paste with higher stiffness exhibits smaller autogenous shrinkage.

The addition of supplementary materials will dramatically affect the internal relative humidity and stiffness of cement paste and further change the autogenous shrinkage [12,13,14,15,16,17]. In the past few years, many studies have been carried out on the effect of silica fume [12,13], fly ash [14,15] and blast furnace slag [16,17] on autogenous shrinkage. In those studies, the most concern is given to the cement mortar and concrete. Few experimental results about the autogenous shrinkage of paste can be found in the literature. Mortar and concrete are assumed to consist of two phases—i.e., sand/aggregate particles and the cement paste matrix, while the autogenous shrinkage only takes place in the cement paste. The study of autogenous shrinkage of cement paste is very important for a better understanding of that of mortar and concrete. Another limitation of the existing studies is that most of them are only about one type of supplementary material and the water–binder ratio of mixtures concerned in different studies always varies [12,13,14,15,16,17,18,19,20]. Therefore, it is hard to compare the effects of different supplementary materials on autogenous shrinkage based on the available results from the literature. 

In order to better study the effect of different supplementary materials on the autogenous shrinkage of cement paste, which is useful for the better prediction of autogenous shrinkage of high-performance concrete that always contains supplementary materials, measurements of final setting time, internal relative humidity, chemical shrinkage, compressive strength and autogenous deformation of cement paste with various supplementary materials are presented and discussed in this paper. Portland cement, silica fume, fly ash and blast furnace slag are investigated. The influence of water–binder ratios is also studied. The results of this study contribute to a better understanding of the autogenous shrinkage of plain and blended cement pastes.

## 2. Materials and Experiments

The materials used in this study are Portland cement (CEM I 42.5N), silica fume, fly ash, blast furnace (BFS) slag cement (CEM III/B 42.5N) and de-ionized water. The mineral composition of Portland cement is presented in Table 1. The chemical compositions of the materials are given in Table 2.

**Figure 1:**
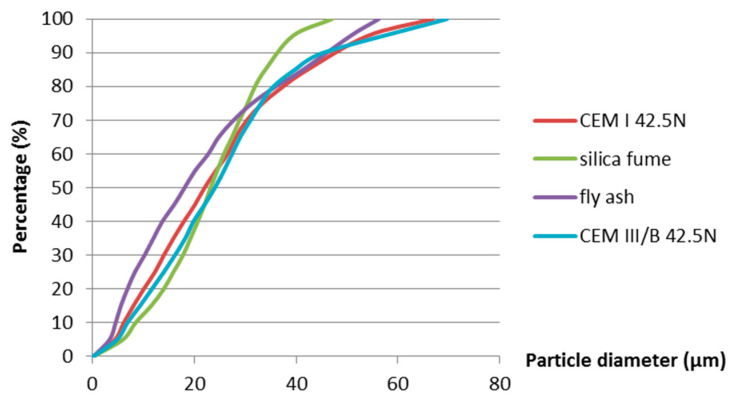
Particle size distribution of materials powders.

Figure 1 shows the particle size distribution curves of Portland cement, silica fume, fly ash and BFS cement measured by laser diffraction. The silica fume used in this paper is commercial dry-densified silica fume. The mean particle size, *D50*, of Portland cement, silica fume, fly ash and BFS cement is 22 µm, 23 µm, 18 µm and 24 µm, respectively.

The experimental series considered in this study comprised eight mixtures. Portland cement pastes (CEM I 42.5N) serve as references. The silica fume and fly ash dosages in blended mixtures were 10% and 30% by weight of the binder, respectively. BFS cement (CEM III/B 42.5N) was also used, in which clinker accounted for 34% by mass. The water/binder ratios were 0.3 and 0.4. The mixture compositions are listed in Table 3. Cement paste was mixed in an epicyclic Hobart mixer (HL120, Hobart, Offenburg, Germany). De-ionized water was mixed with the admixtures and added in two steps to ensure homogeneity. After the addition of cement binder in the water allowed 60 s for the absorption of the water before it was mixed at a slow speed for 30 s. Then, the mixer was stopped for 30 s and, during this time any paste that may have collected on the side of the bowl was scraped down into the batch. Start the mixer at medium speed and mix for 60 s.

### 2.1. Final Setting Time

**Figure 2:**
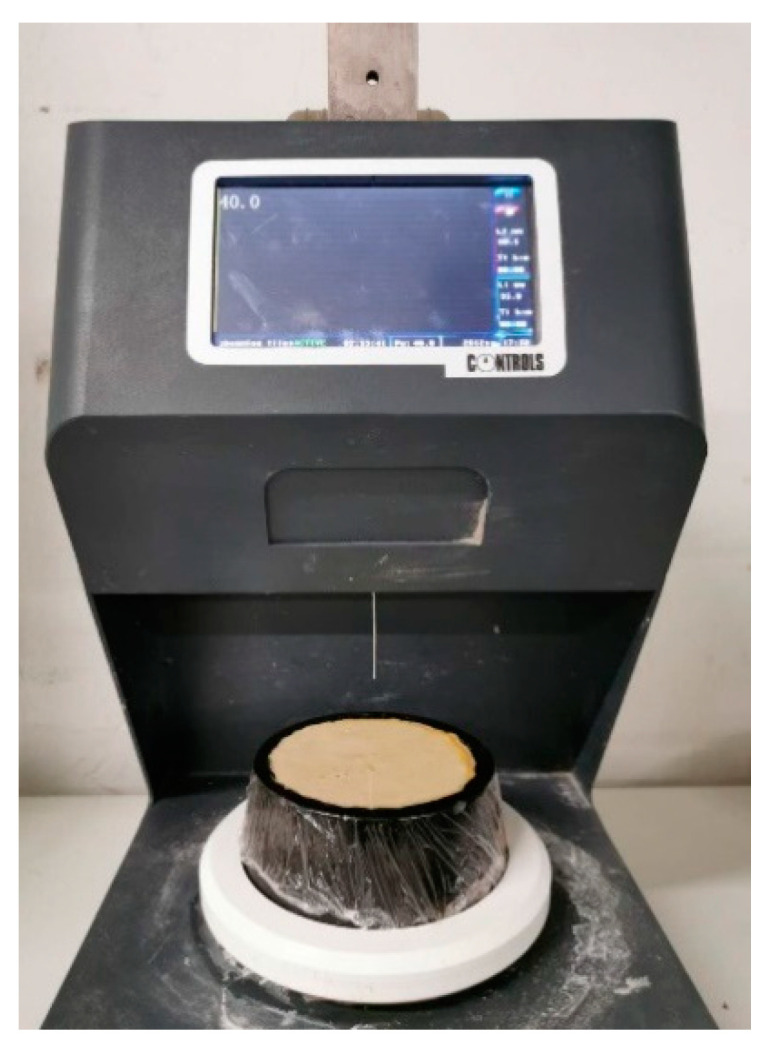
Automatically recording Vicat apparatus.

The final setting time was taken as the starting time of the measurement of autogenous shrinkage. In this study, the final setting time was determined by the Vicat method according to standard NEN-EN 196-3:2005. An automatically recording Vicat apparatus (Controls VICATMATIC 2, CONTROLS Group, Milan, Italy) was used (Figure 2).

### 2.2. Chemical Shrinkage

**Figure 3:**
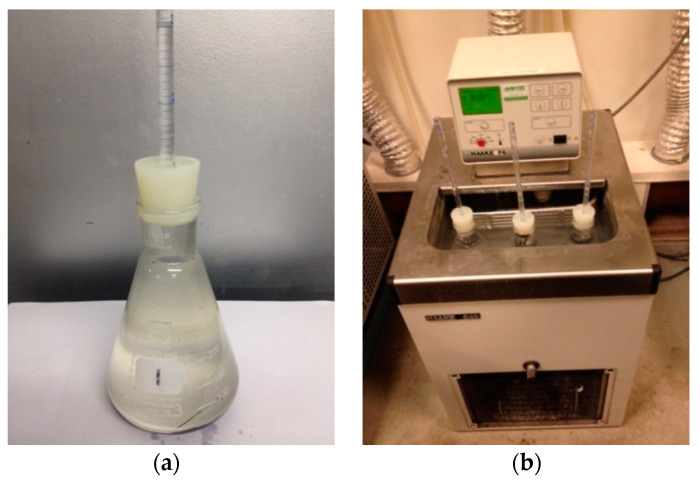
Equipment for chemical shrinkage measurement. (**a**) Glass Erlenmeyer flask with graduated tubes; (**b**) Constant-temperature water bath.

The measurement of chemical shrinkage was according to the ASTM C1608 [21]. About 50 g of freshly mixed cement paste was put in an Erlenmeyer flask (Figure 3a), with a capacity of 250 mL. The thickness of the cement paste sample was about 8 mm. After the cement paste was covered with a thin layer of de-ionized water, the Erlenmeyer flask was filled with paraffin oil and sealed with a rubber stopper encasing a graduated tube with a total volume of 5 ± 0.1 mL. The flask was then immersed in a constant temperature water bath at 20 °C (Figure 3b). The first measurement was performed 30 min after the immersion of the samples in the water bath. Measurements were performed for 7 days. The chemical shrinkage is presented as the volume decrease in the paste per gram of cement and supplementary materials. For each measurement, two specimens were tested.

### 2.3. Internal Relative Humidity

**Figure 4:**
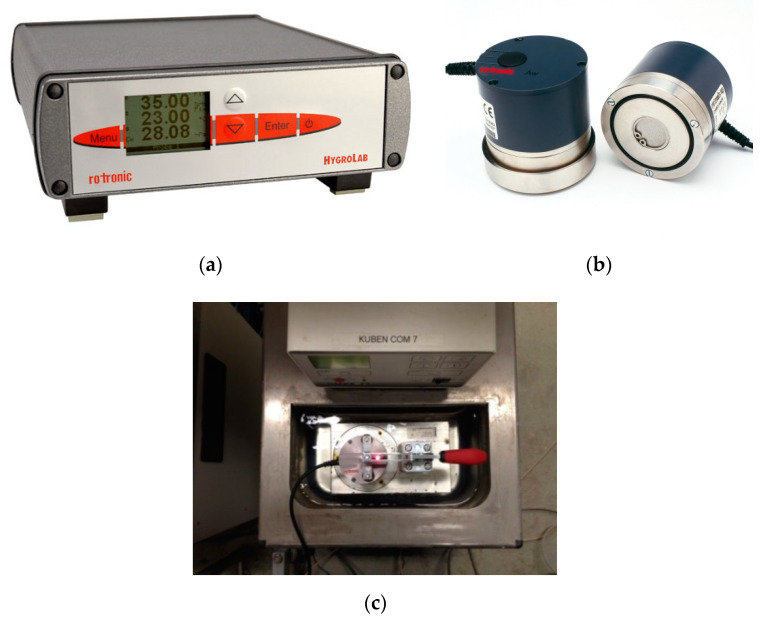
Apparatus for internal relative humidity measurement; (**a**) Rotronic HygroLab C1; (**b**) HC2-AW RH station probes; (**c**) Top view of temperature-controlled water bath.

The development of the internal relative humidity in the paste was measured by Rotronic HygroLab C1 (HygroLab C1, Rotronic, Bassersdorf, Switzerland) (Figure 4a) equipment with two HC2-AW RH station probes (HC2-AW-USB, Rotronic, Bassersdorf, Switzerland) with an accuracy ± 0.5% (Figure 4b). The RH probes were placed in a temperature-controlled water bath at 20 °C (Figure 4c). The RH probes were calibrated using saturated salt solutions with known constant RH in the range of 65–95%. After calibration, the freshly mixed cement pastes were cast in two plastic containers and then put into the measuring chambers. The RH in the samples and the temperature were recorded every 2 min. The duration of the test was 7 days.

### 2.4. Compressive Strength

Compressive strength tests were carried out after 1, 3 and 7 days of sealed curing on cement paste cubes, 40 × 40 °C 40 mm^3^. The cubes were cured in a sealed condition at 20 °C. At least three specimens were tested for each measurement.

### 2.5. Autogenous Deformation

**Figure 5:**
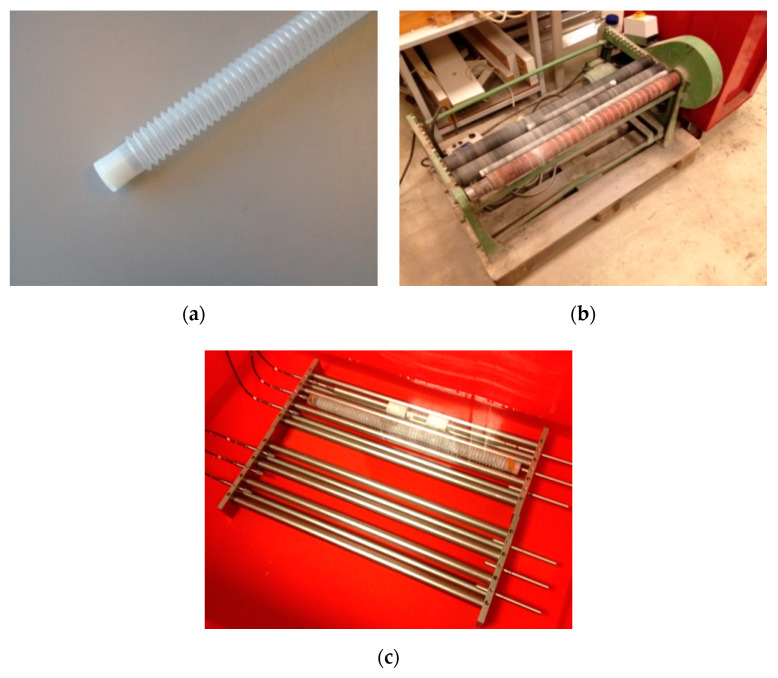
Setup for the autogenous shrinkage measurement; (**a**) Corrugated plastic tube; (**b**) Rotation machine; (**c**) Frame.

The cement paste was cast under vibration into tight plastic molds (low-density polyethylene plastic, LDPE), which were corrugated to minimize restraint on the paste (Figure 5a). The length of the samples was approximately 430 mm and the diameter was 29 mm. Measurement of autogenous shrinkage started after the final setting time when a solid skeleton of cement paste formed. Before that time, the specimens were placed on a rotation machine (self-manufactured) at a speed of 10 rpm to avoid bleeding (Figure 5b). The specimens were placed in a frame and immersed into a temperature-controlled glycol bath at 20 ± 0.1 °C. A top view of the frame is shown in Figure 5c. The frame consisted of two steel plates joined rigidly by six solid invar rods (diameter 20mm). Each specimen was longitudinally supported by two parallel rods attached to the steel plates. The specimens were gripped by screws at one end, while the rest could slide freely on the rods, which were lubricated by the glycol bath. The longitudinal deformation was measured at the free end by a TRANS-TEK 350-000 displacement transducer (Series 350, TRANS-TEK, Ellington, United States). Three samples were tested in the frame simultaneously, with a measurement accuracy of ± 5 µm. Length changes were recorded every 5 min.

## 3. Results and Discussions

### 3.1. Final Setting Time

**Figure 6:**
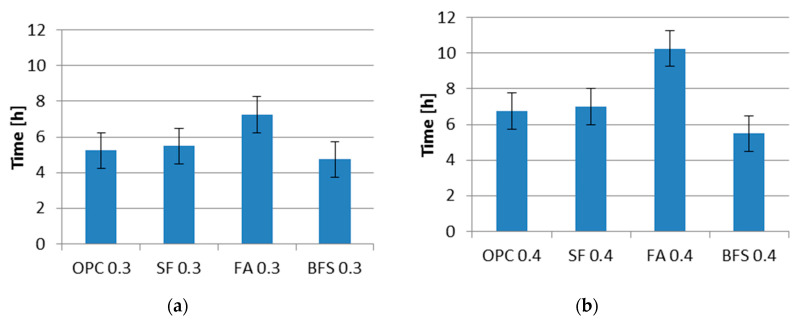
Final setting time of cement pastes with different supplementary materials. Code: See Table 3; (**a**) Water–cement ratio of 0.3; (**b**) Water–cement ratio of 0.4.

In Figure 6 the final setting times of cement pastes with water–binder ratios of 0.3 and 0.4, cured at 20 °C, are shown. From Figure 6 it can be seen that the final setting time is longer for pastes with higher water–binder ratios for all kinds of cement paste. Fly ash cement paste has a longer setting time than other cement pastes with the same water–binder ratio.

Some authors [22] reported that silica fume accelerates the reaction of C_3_A and C_3_S during the first hours of hydration. However, as shown in Figure 6, the final setting times seem hardly influenced by the addition of silica fume. In these experiments, silica fume was added in dry densified form. The silica fume used in other studies was often in slurry form. The phenomenon of accelerated hydration reported by other authors is due to the extreme fineness of silica fume they used. The commercial dry densified silica fume used in this paper exists primarily in the form of clusters of spheres. The influence on the hydration process and final setting time is not as pronounced as that of silica fume added in slurry form. That coarse silica fume does not significantly influence the final setting time was also found by Rao [23].

The addition of fly ash increases the final setting time of fly ash cement paste compared to that of ordinary Portland cement paste. This result is in line with the finding of Berg and Kukko [24]. Compared with Portland cement, fly ash contains a higher amount of inactive minerals, such as quartz and mullite [25,26]. As a consequence of this, the fly ash reacts slower than cement at an early age and results in a longer final setting time.

Figure 6 shows that the final setting time of CEM III/B 42.5N is shorter than that of CEM I 42.5N. The shorter setting time of BFS cement paste has also been observed by Xiao et al. [27]. This, however, is contradictory to the common understanding that the setting time will increase with the addition of BFS. If the Portland cement and BFS cement are made with the same kind of clinker, the lower clinker content in BFS cement results in longer final setting time. According to the producer, CEM I 42.5N and CEM III/B 42.5N used in this study are made with different kinds of clinker. CEM III/B 42.5N made with higher activity clinker may have shorter final setting time than that of CEM I 42.5N made with lower activity clinker.

### 3.2. Chemical Shrinkage

**Figure 7:**
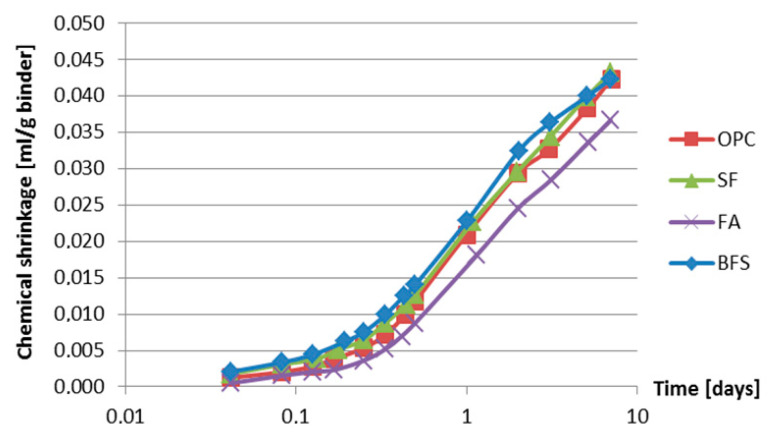
Chemical shrinkage as a function of age.

Measured chemical shrinkage of four different types of cement is displayed in Figure 7. The mixture compositions of these cements are listed in Table 4.

Chemical shrinkage of fly ash cement paste is lower than that of other mixtures in the first week. Chemical shrinkage is proportional to the degree of hydration [28,29]. Compared with Portland cement, fly ash contains a higher amount of inactive minerals, such as quartz and mullite. As a consequence of this the fly ash reacts slower than cement and results in smaller chemical shrinkage at early age [30].

Chemical shrinkage of the BFS cement paste develops faster than that of Portland cement paste in the first 3 days. These results are in line with the findings of Bentz [31]. According to [31] the density of hydration products of Portland cement and BFS cement paste is similar. The density of BFS particles is lower than that of Portland cement. The lower density of BFS results in a bigger volume change between unhydrated BFS and hydration products.

### 3.3. Internal Relative Humidity

**Figure 8:**
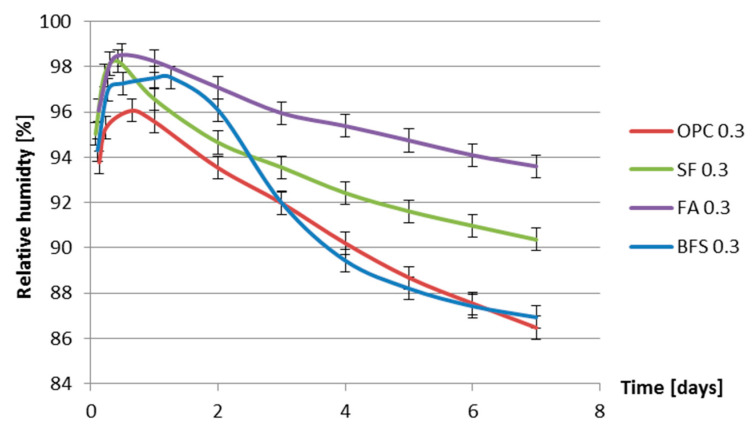
Internal relative humidity vs. age for different cement pastes with water–binder ratio of 0.3.

**Figure 9:**
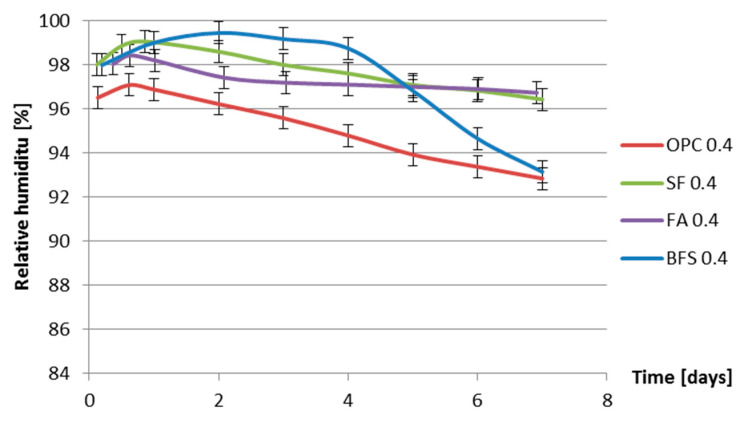
Internal relative humidity vs. age for different cement pastes with water–binder ratio of 0.4.

The internal relative humidity of the cement pastes was measured for a period of 7 days. For each series two specimens were tested. The difference between measured internal relative humidity of two specimens was less than 1%. The development of internal relative humidity with hydration time is provided in Figure 8 and Figure 9.

Figure 8 and Figure 9 show that the relative humidity of fly ash cement paste is higher than that of ordinary Portland cement paste with the same water–binder ratio. The higher relative humidity of fly ash cement paste is in accordance with the findings of Varga et al. [32]. Fly ash reacts slower than Portland cement. At the same, curing age the non-evaporable water content in fly ash cement pastes is lower than that in ordinary Portland cement pastes with the same water–binder ratio. More water is present in the pore structure of fly ash cement paste which results in higher relative humidity.

From Figure 8 and Figure 9, it can be found that the moment that the relative humidity of BFS cement paste starts to drop significantly is later than that of Portland cement paste with the same water-binder ratio. For BFS cement paste with water-binder ratio 0.3 the relative humidity starts to drop 0.6 day later than that in the Portland cement paste with water-binder ratio 0.3. For BFS cement paste with water–binder ratio 0.4, the relative humidity starts to drop even 3.5 days later than that of Portland cement paste with water-binder ratio 0.4. A similar result can be found in Lura’s thesis [6]. In [6], the relative humidity of BFS cement paste with water–binder ratio 0.37 starts to drop 1 day later than in the Portland cement paste. The later starting moment of the RH drop of BFS cement paste can be attributed to the low activity of BFS after final setting. According to Taylor [33], the hydration rate of BFS at early age is much slower than that of Portland cement. BFS cement (CEM III/B 42.5N) used in the test series contains BFS (66% by mass) and Portland clinker (34% by mass). The large amount of low active BFS in CEM III/B 42.5N results in the lower water consumption of BFS cement during the first few days of hydration and a later starting moment of RH drop of BFS cement paste.

### 3.4. Compressive Strength

**Figure 10:**
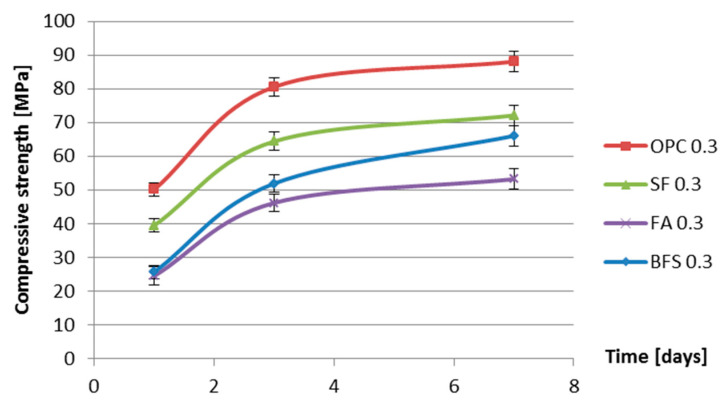
Compressive strength vs. age for different cement pastes with water–binder ratio of 0.3.

**Figure 11:**
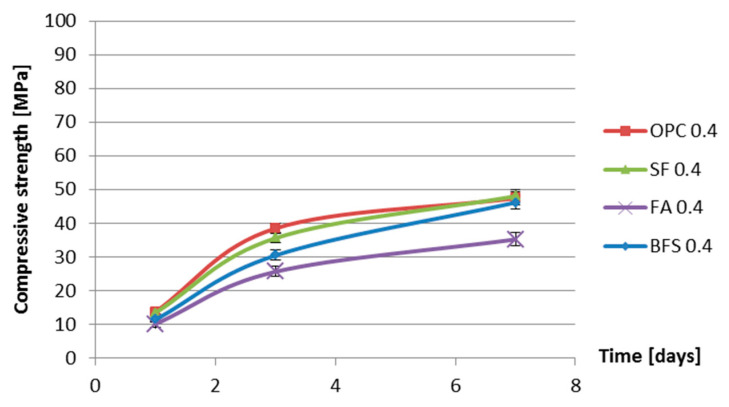
Compressive strength vs. age for different cement pastes with water–binder ratio of 0.4.

Figure 10 and Figure 11 show the compressive strength as a function of age of four cement pastes with water–binder ratios of 0.3 and 0.4.

From Figure 10 and Figure 11 it can be seen that, during the first 7 days, the compressive strengths of the samples with silica fume are lower than those of the samples without silica fume. This is different from the common understanding that the addition of silica fume will increase the strength of concrete. Houssam and Tahar [34] pointed out that the increase in the strength of mortar and concrete with the incorporation of silica fume is due to the improvement of the aggregate–matrix bond. For the silica fume pastes, in the absence of the interfacial transition zone, there is no substantial strengthening effect.

### 3.5. Autogenous Deformation

**Figure 12:**
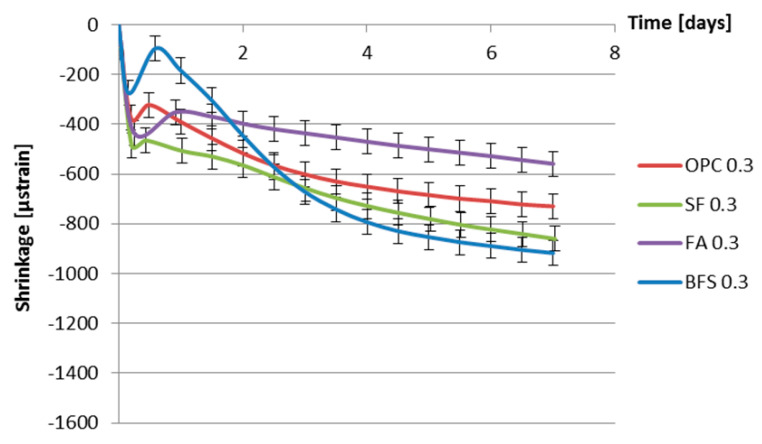
Autogenous deformation vs. age for different cement pastes with water–binder ratio of 0.3 (starting time: final setting time).

**Figure 13:**
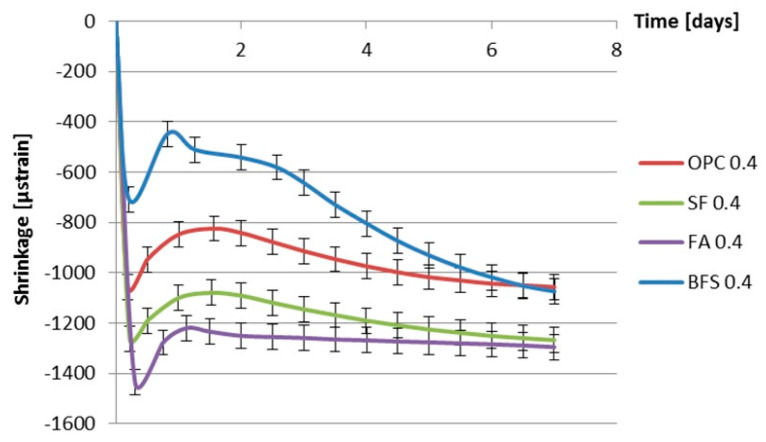
Autogenous deformation vs. age for different cement pastes with water–binder ratio of 0.4 (starting time: final setting time).

Figure 12 and Figure 13 show the measured autogenous deformations as a function of age of different cement pastes with a water–binder ratio of 0.3 and 0.4. Three samples were tested simultaneously.

**Figure 14:**
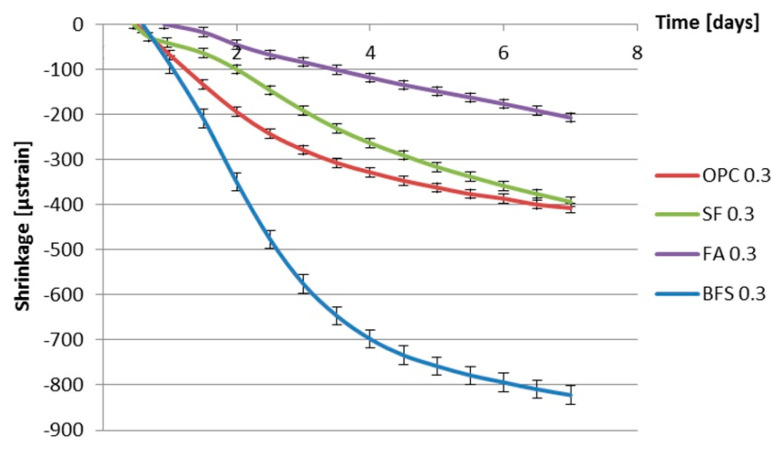
Autogenous deformation vs. age for different kinds of cement paste with water–binder ratio of 0.3 (starting time: after early-age swelling).

**Figure 15:**
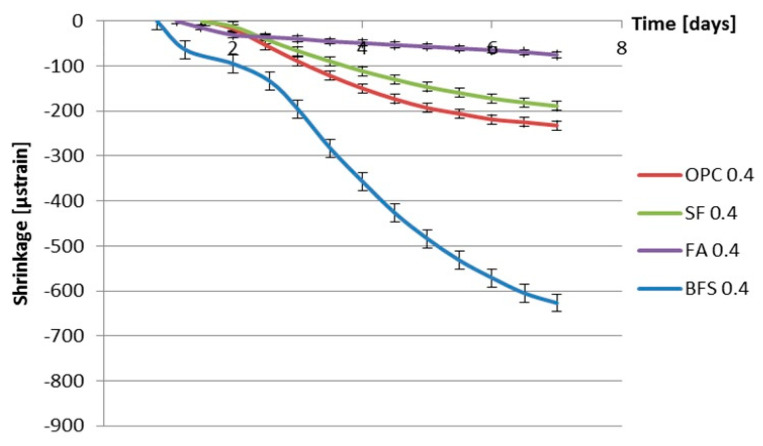
Autogenous deformation vs. age for different kinds of cement paste with water–binder ratio of 0.4 (starting time: after early-age swelling).

From Figure 12 and Figure 13, a fast shrinkage can be seen after final setting. After a short period of swelling, the specimens shrink steadily. According to some researchers [35,36,37,38], taking the final setting time as the starting point of autogenous shrinkage is questionable. The starting time of autogenous shrinkage is roughly equal to the setting time but is not necessarily identical with it [39]. A lot of researchers start counting autogenous shrinkage from “time-zero” which is defined as the duration between this instant when the water comes in contact with cement and the time at which the concrete develops sufficient structure to enable tensile stress transfer through the concrete [40,41]. According to Bjøntegaard [42], the time when the maximum (macroscopically) swelling is observed can be taken as the starting time of autogenous shrinkage (“time-zero”). In this section, the steady shrinkage after maximum (macroscopically) observed swelling is considered as autogenous shrinkage of the cement pastes as shown in Figure 14 and Figure 15. It can be found that BFS cement paste has the highest autogenous shrinkage amongst all the mixtures with the same water–binder ratio, while the autogenous shrinkage of cement paste with the incorporation of fly ash is the lowest.

### 3.6. Discussion of the Effect of Supplementary Materials on Autogenous Shrinkage

Figure 14 and Figure 15 show that, in the first 7 days, the addition of fly ash leads to smaller autogenous shrinkage of cement paste compared with that of ordinary Portland cement paste with the same water–binder ratio. These results are in line with findings of Tangtermsirikul [14]. The lower activity of fly ash compared to ordinary Portland cement is considered the major reason for the smaller autogenous shrinkage of fly ash cement paste [30]. The low activity of fly ash led to slower hydration and resulted in a slower decrease in the internal relative humidity and smaller shrinkage at an early age.

Figure 14 and Figure 15 also show that, in the first 7 days, the addition of silica fume does not lead to bigger autogenous shrinkage of cement paste compared with that of ordinary Portland cement paste with same water–binder ratio. This result is contradictory to the finding reported by Jensen and Hansen [13], that the addition of silica fume significantly increases the autogenous shrinkage of cement paste. The silica fume used by Jensen and Hansen is in slurry form, which has large specific surface area and high activity at early age. High activity of silica fume results in larger chemical shrinkage and a bigger drop in internal relative humidity, which results in larger autogenous shrinkage of cement paste [13]. The silica fume used in this paper is commercial dry densified silica fume. The mean particle sizes of silica fume are not significantly smaller than Portland cement (as shown in Figure 1). The activity of dry densified silica fume at early age is not as high as that of silica fume in slurry form. The drop in internal relative humidity of cement paste does not increase with the addition of dry densified silica fume (as shown in Figure 8 and Figure 9) and, therefore, the autogenous shrinkage of silica fume cement paste is not significantly bigger than that of ordinary Portland cement paste with same water–binder ratio.

The measured autogenous shrinkage of BFS cement paste is much bigger than that of Portland cement paste with the same water–binder ratio. According to Chan et al. [16], the autogenous deformation of concrete with 40% BFS is significantly higher than that of concrete without BFS. When the BFS content is higher, the autogenous deformation decreases slightly, but it still remains higher than that of concrete without BFS. The bigger autogenous shrinkage of BFS cement paste has two reasons. First, the drop in internal relative humidity of BFS cement paste is larger than that of ordinary Portland cement paste with the same water–binder ratio, as shown earlier in Figure 8 and Figure 9. Second, the stiffness of BFS cement paste is lower than that of Portland cement paste with the same water–binder ratio at the same curing age, as indicated by the strength results shown in Figure 10 and Figure 11. Similar results are also found by Lura [6] and Ekaputri [43]. According to Ishida et al. [44], BFS cement pastes have a finer pore structure than Portland cement pastes. Finer pores of BFS cement paste result in a smaller radius of air-water meniscus and larger internal driving force of autogenous shrinkage—e.g., capillary tension.

## 4. Concluding Remarks

In order to study the effect of supplementary materials on the autogenous shrinkage of cement paste, measurements of final setting time, internal relative humidity, chemical shrinkage, compressive strength and autogenous deformation of cement pastes are presented and discussed in this paper. Pure Portland cement paste and three kinds of cement paste with different supplementary material—i.e., silica fume, fly ash and blast furnace slag—were considered. Water–binder ratios of these cement pastes are 0.3 and 0.4. The following conclusions can be drawn:

### 4.1. Final Setting Time

The final setting times seem hardly influenced by the addition of silica fume. The influence of the commercial dry densified silica fume used in this paper on the hydration process and final setting time is not as pronounced as that of silica fume added in slurry form. The addition of fly ash increases the final setting time of fly ash cement paste compared to that of ordinary Portland cement paste.

### 4.2. Chemical Shrinkage

Up to seven days, the chemical shrinkage of the cement paste with fly ash was much smaller than that of Portland cement paste. Chemical shrinkage of the BFS cement paste develops faster than that of Portland cement paste in the first 3 days.

### 4.3. Internal Relative Humidity

The moment that the relative humidity of BFS cement paste starts to drop significantly is later than that of Portland cement paste with the same water–binder ratio. The later start of the RH drop of BFS cement paste can be attributed to the low activity of BFS in CEM III/B 42.5N after the final setting time.

### 4.4. Compressive Strength

The compressive strength of the samples with silica fume is lower than of the samples without silica fume. This is different from the common understanding that the addition of silica fume will increase the strength of concrete. The increasing strength of silica fume concrete is caused by the improvement of the aggregate–matrix bond. For the silica fume pastes, in the absence of the interfacial transition zone, there is no substantial strengthening effect.

### 4.5. Effect of Supplementary Materials on Autogenous Shrinkage

The type of cement has a significant effect on autogenous shrinkage. The addition of fly ash resulted in smaller autogenous shrinkage of cement paste compared with that of ordinary Portland cement paste with the same water–binder ratio. The low activity of fly ash is considered the major reason for this.

The addition of commercial dry densified silica fume does not lead to bigger autogenous shrinkage of cement paste compared with that of ordinary Portland cement paste with the same water–binder ratio. The activity of dry densified silica fume at early age is not as high as that of silica fume in slurry form. The drop in internal relative humidity of cement paste does not increase with the addition of dry densified silica fume and the autogenous shrinkage of silica fume cement paste is not significantly bigger than that of ordinary Portland cement paste with same water–binder ratio. The measured autogenous shrinkage of BFS cement paste is much bigger than that of Portland cement paste with the same water–binder ratio. The lower elastic modulus and a larger drop in relative humidity of BFS cement paste are considered as the two major reasons.

### 4.6. Prospects

The results presented in this paper are very useful for the prediction of autogenous shrinkage of concrete made by different supplementary materials. Concrete consists of two parts: shrinking cement paste matrix and non-shrinking sand/aggregate particles. In future studies, with the knowledge of autogenous shrinkage of cement paste, the autogenous shrinkage of concrete can be predicted by taking the restraining effect of non-shrinking sand/aggregate particles into consideration.

## Figures and Tables

**Table 1:** Mineral composition of Portland cement (% by weight).

Phase	Weight (%)
C_3_S	67.1
C_2_S	5.9
C_3_A	7.8
C_4_AF	9.6
Other	9.6

**Table 2:** Chemical composition of materials (% by weight).

Chemical Composition	CEM I 42.5N	Silica Fume	Fly Ash	CEM III/B 42.5N
SiO_2_	20.36	97.2	48.4	30.61
Al_2_O_3_	4.96	0.51	31.4	10.58
CaO	64.4	0.39	7.14	45.52
Fe_2_O_3_	3.17	0.18	4.44	1.42
K_2_O	0.64	1.04	1.64	0.58
MgO	2.09	-	1.35	7.33
SO_3_	2.57	0.26	1.18	2.66
Na_2_O	0.14	-	0.72	0.31
Total	98.33	99.58	96.7	99.01

**Table 3:** Mixture composition of Portland cement paste and blended cement paste (% by weight).

Name	CEM I 42.5N (%)	CEM III/B 42.5N (%)	Silica Fume (%)	Fly Ash (%)	Water/Binder (w/b)
OPC 0.3	100	0	0	0	0.3
OPC 0.4	100	0	0	0	0.4
SF 0.3	90	0	10	0	0.3
SF 0.4	90	0	10	0	0.4
FA 0.3	70	0	0	30	0.3
FA 0.4	70	0	0	30	0.4
BFS 0.3	0	100	0	0	0.3
BFS 0.4	0	100	0	0	0.4

**Table 4:** Mixture composition of Portland cement and blended cement (% by weight).

Name	CEM I 42.5N (%)	CEM III/B 42.5N (%)	Silica Fume (%)	Fly Ash (%)
OPC	100	0	0	0
SF	90	0	10	0
FA	70	0	0	30
BFS	0	100	0	0
